# Progress testing in the medical curriculum: students’ approaches to learning and perceived stress

**DOI:** 10.1186/s12909-015-0426-y

**Published:** 2015-09-11

**Authors:** Yan Chen, Marcus Henning, Jill Yielder, Rhys Jones, Andy Wearn, Jennifer Weller

**Affiliations:** 1 Centre for Medical and Health Sciences Education, University of Auckland, Building 599, Level 12, Auckland City Hospital, Private Bag 92019, Auckland, 1142 New Zealand; 2 Medical Programme Directorate, University of Auckland, Auckland, New Zealand; 3 Te Kupenga Hauora Māori, University of Auckland, Auckland, New Zealand; 4 Clinical Skills Centre, University of Auckland, Auckland, New Zealand

## Abstract

**Background:**

Progress Tests (PTs) draw on a common question bank to assess all students in a programme against graduate outcomes. Theoretically PTs drive deep approaches to learning and reduce assessment-related stress. In 2013, PTs were introduced to two year groups of medical students (Years 2 and 4), whereas students in Years 3 and 5 were taking traditional high-stakes assessments. Staged introduction of PTs into our medical curriculum provided a time-limited opportunity for a comparative study. The main purpose of the current study was to compare the impact of PTs on undergraduate medical students’ approaches to learning and perceived stress with that of traditional high-stakes assessments. We also aimed to investigate the associations between approaches to learning, stress and PT scores.

**Methods:**

Undergraduate medical students (*N* = 333 and *N* = 298 at Time 1 and Time 2 respectively) answered the Revised Study Process Questionnaire (R-SPQ-2F) and the Perceived Stress Scale (PSS) at two time points to evaluate change over time. The R-SPQ-2F generated a surface approach and a deep approach score; the PSS generated an overall perceived stress score.

**Results:**

We found no significant differences between the two groups in approaches to learning at either time point, and no significant changes in approaches to learning over time in either cohort. Levels of stress increased significantly at the end of the year (Time 2) for students in the traditional assessment cohort, but not in the PT cohort. In the PT cohort, surface approach to learning, but not stress, was a significant negative predictor of students’ PT scores.

**Conclusions:**

While confirming an association between surface approaches to learning and lower PT scores, we failed to demonstrate an effect of PTs on approaches to learning. However, a reduction in assessment-associated stress is an important finding.

## Background

Progress tests (PTs) in medical programmes are designed to assess applied medical knowledge at the level of a new graduate and are administered to all students across all years of a programme [1, 2]. PTs are intended to discourage students from preparing specifically for a test and then ‘discarding’ that knowledge. Applied medical knowledge from any stage of the curriculum can appear in a PT; therefore PTs should promote meaning-orientated learning and also foster long-term knowledge retention, while reducing superficial learning strategies such as rote learning and ‘cramming and dumping’ [1, 3–5]. PT performance is also not affected by small changes in curriculum delivery as each test is drawn from a bank of questions which cover the major areas of basic, clinical and behavioural science, and public health, expected of a graduating doctor [6].

Due to their longitudinal nature, PTs are intended to demonstrate knowledge growth as students advance in their undergraduate study. PTs also provide comprehensive feedback to students so they can identify gaps in their knowledge base, which facilitates self-directed learning. PTs could potentially reduce levels of anxiety and stress in students as each PT judgement is made at multiple step-points in the programme rather than at a single end of year examination [3, 7].

### Progress testing and students’ approaches to learning

At least three different approaches to learning can be adopted by students [8, 9]. A surface approach features rote learning and is driven by work overload, time pressure or extrinsic motivation such as the fear of failure. In contrast, a deep approach to learning features the desire to learn the meaning and applicability of concepts and is often driven by intrinsic motivation. A third type of approach to learning, the achievement, or strategic approach is used when students are driven by extrinsic motivation such as the rewards associated with high marks but have minimal input of time. However, unlike deep and surface approaches, there is considerable disagreement over whether this separate ‘strategic’ approach exists possibly due to its overlap with the surface approach to learning [10, 11].

The type of assessment influences students’ approaches to learning [12]. A recent review shows that formative assessment is likely to facilitate students’ deep approaches to learning, whereas summative assessment is more likely to drive students to surface approaches [13]. In line with this claim, PTs should foster deep approaches to learning while reducing the tendency for students to incorporate surface approaches to learning [3, 5]. However, the impact of PTs on student learning can vary with context and curriculum structure [14], and there are inconsistent findings to support the claim that PTs encourage students to adopt deep learning strategies. For instance, van Berkel et al. [7] found that PTs are more likely to penalise a surface approach, but this does not equate to increasing the use of a deep approach. Moreover, this study examined only one time point and not changes in approaches over time. Blake et al. [4] suggested that PTs can maintain a sense of objectivity around testing but also create the opportunity for the development of deep learning by providing students with accurate and comprehensive feedback culminating in a mastery of knowledge. However, Blake et al.’s longitudinal study found no evidence to suggest that students changed their learning approach as a result of PTs. Further investigation is needed to verify such claims that PTs cultivate deep approaches to learning.

### Progress testing and perceived stress

In comparison to their non-medical counterparts, lower levels of psychological wellbeing have been reported by medical students across different education systems [15–18]. For instance, medical students are more likely to experience high levels of stress, which could be related to exam preparations and a lack of guidance and support from the medical school during transitions between pre-clinical and clinical years [19]. These negative psychological outcomes are also likely to persist into the students’ careers as health practitioners, as evidenced in high levels of work-related distress and burnout in doctors [20].

Contrary to traditional, high-stakes assessment, PTs could reduce the levels of stress toward examination preparation. For instance, van der Vleuten’s [3] study found that students paid little attention to PTs in terms of exam preparation and they did not feel under pressure going into the tests. Also, the impact of failing a single PT was perceived as less stressful, relative to traditional end of year examinations, as students’ applied medical knowledge is assessed based on all successive PTs sat across their undergraduate years. However, the relatively low PT scores that students obtain in the first few years are not as rewarding as the potentially high grades they receive from traditional summative examinations [7]. This may potentially cause some levels of distress for medical students, as they enter with high academic credentials and are used to performing at the high end of peer cohorts. Indeed, Mattick and Knight [21] carried out semi-structured interviews with Year 2 undergraduate medical students and found that PTs could be a barrier to high-quality learning for students who become excessively anxious about sitting them. Therefore, the impact of PTs on medical students’ perceived stress should be of interest to medical educators.

### Associations between academic performance, approaches to learning, and perceived stress

Both approaches to learning and the assessment-related anxiety and stress are predictors of students’ academic performance [22]. Snelgrove and Slater [23] reported positive correlations between deep approaches to learning and sociology students’ Grade Point Average (GPA) and negative correlations between surface approaches to learning and nursing students’ GPA. In contrast, Duff [24] found both deep and surface approaches to learning were negatively associated with course grades of a Master of Business Administration programme. Furthermore, deep approaches to learning account for unique variance in students’ academic performance above and beyond individual personality and intelligence [25]. On the other hand, the levels of anxiety and stress consistently predict lower levels of academic performance [22]. However, most of the previous research used students’ GPA as a measure of academic performance. The relationship between approaches to learning, students’ stress, and other measures of academic performance, such as students’ PT scores, needs to be examined. In addition, the potential influence of age and gender on the association between approaches to learning and PT scores should be considered as both factors relate to how students approach learning [26].

PTs were introduced to the University of Auckland’s Bachelor of Medicine and Bachelor of Surgery programme (MBChB) programme in 2013, with progressive roll-out for different year cohorts over a period of time. The staged introduction of PTs provided us with a time-limited opportunity to compare PTs with traditional assessments in a single medical programme.

### Research questions

The overall aim of our research is to explore the effect of PTs on approaches to learning and stress and to compare this with traditional high-stakes assessments. However, we also need to consider the potential influence of other factors such as age and gender [25]. We proposed the following, specific research questions:

1. Do PTs increase deep approaches to learning and/or decrease surface approaches to learning compared with traditional assessments?
2. Do PTs elicit less self-reported stress in students than traditional assessments?
3. Do deep or surface approaches to learning correlate with self-reported stress or PT scores?

## Methods

The University of Auckland Human Participants Ethics Committee approved this study (Reference number 9758). Participation was voluntary and informed consent was obtained from each participant.

### Study setting

Medical education at The University of Auckland lasts six years, consisting of generic health science courses in the first year, followed by admission into the MBChB programme from Year 2 onwards via academic performance, a reasoning test and an interview. About a quarter of the medical student cohort are graduates who enter the programme at Years 2. The curriculum in Years 2 and 3 focuses on biomedical science knowledge, clinical and professional skills, with limited exposure to clinical settings. PTs are used in combination with end of module assessments, and contribute 15 % in Year 2, and 40 % in Year 3 to final marks for applied medical knowledge. Years 4 and 5 are focused on applying medical knowledge in clinical settings. In Year 6 of the programme students take on some responsibility for clinical care in preparation for their first postgraduate year, undertaking tasks that are similar to a new graduate but with more supervision and some limitations. In Years 4–6, PTs contribute 100 % to the final marks for applied medical knowledge. Therefore, it is possible to fail the year based on PT grades.

PTs were first introduced to two student cohorts (Years 2 and 4 in 2013), and it has since been progressively rolled out to all year groups. The decision on the staged roll-out for different year groups was consistent with curriculum changes in the different years introduced in in 2013. This created a quasi-experimental comparison based on a naturally occurring educational circumstance. Table 1 provides further detail about the differences between the PT and traditional groups.

**Table 1:** Participant group composition and survey response rate

Group	Year in programme	Response rate (Time 1 & Time 2)	Assessments
Progress testing (PT)	Year 2 (pre-clinical)	46 % & 33 %	PTs & end of module tests
	Year 4 (clinical)	42 % & 47 %	PTs, workplace-based assessments (WBA) & objective structured clinical examinations (OSCE)
Traditional	Year 3 (pre-clinical)	43 % & 25 %	End of year examinations & end of module tests
	Year 5 (clinical)	21 % & 34 %	End of year examinations, workplace-based assessments (WBA) & objective structured clinical examinations (OSCE)

### Participants

In 2013, students who were enrolled in the MBChB programme (Year 2 to Year 5) at the University of Auckland received an invitation to participate in an online survey at two time points, corresponding to the second PT (PT2) in July and the third PT(PT3) in October^1^. Year 2 and Year 4 students took PTs at both time points, whereas Year 3 and Year 5 students sat traditional end of year assessments. Based on assessment type, students were divided into two cohorts: PT cohort (Year 2 and Year 4) and traditional assessment cohort (Year 3 and Year 5).

### Survey administration

The survey was distributed online via SurveyGizmo (an Internet-based survey tool) and remained open for two weeks after each of the two sequential PTs in July and October 2013. Participation was voluntary and students were invited and reminded by email.

### Measurements

The online survey consisted of a series of demographic questions and two validated questionnaires measuring students’ approaches to learning and perceived levels of stress.

#### Approaches to learning

Given the uncertainty around strategic approach to learning, we used The Revised Two Factor Study Process Questionnaire [27] (R-SPQ-2F) to assess students’ approaches to learning. The R-SPQ-2F contains 20 items and is believed to measure two types of approaches to learning (a deep approach and a surface approach) that students adopt in higher education settings. Each item is answered on a five-point Likert scale, ranging from 1 (“this item is never or only rarely true of me”) to 5 (“this item is always or almost always true of me”). Two scores, the sum of 10 different items, were produced to indicate a deep and a surface approach to learning.

#### Levels of perceived stress

The Perceived Stress Scale [28] (PSS) was used to measure the levels of stress that students experienced in the month prior to completing the questionnaire. The PSS contains 10 items and each item is answered on a five-point Likert scale, ranging from 0 (never) to 4 (very often). Four items have their answers reverse scored and a total score is calculated to indicate the levels of stress. Research has evaluated the psychometric properties of the PPS (i.e., internal consistency, test-retest validity) and showed that it is suitable to use for undergraduate medical students [29].

### Analysis

A preliminary check of the internal consistency of the questionnaires was assessed using Cronbach’s alpha, and the distribution of questionnaire scores was also examined. Next, descriptive measures were generated for the main variables involved in the study. In addition, age and gender were appraised as potential factors that may influence the analysis. The main analysis was then conducted using a repeated measures analysis of variance approach. Approaches to learning as the dependent variable was considered across two time periods in terms of whether students took PTs versus those who sat traditional examinations. Perceived stress as the dependent variable was then evaluated. With respect to students in PT cohort, we then generated a Pearson’s zero-order correlation matrix to assess associations between the approaches to learning, perceived stress, and PT scores. To further tease out these associations we conducted a hierarchical regression analysis at each time point with PT score as the dependent variable. Independent variables included in the model were year in programme, age, and gender (step 1) followed by approaches to learning and perceived stress (step 2).

## Results

Survey responses were received from 333 and 298 (Time 1 and Time 2 respectively) of the 864 students surveyed. Response rates per year group and demographics are described in detail in Table 1.

A preliminary analysis to support questionnaire reliability in our cohort showed our two questionnaire measures had acceptable levels of internal consistency [30]. Cronbach’s alpha ranged from .80 to .82 for the deep approach subscale of the R-SPQ-2F, from .79 to .80 for the surface approach subscale of the R-SPQ-2F, and from .87 to .90 for the PSS. Our questionnaire variables were linearly distributed and their residuals were independently and normally distributed, meeting the assumptions to conduct further parametric analysis [31].

### Descriptive statistics and preliminary analyses

Scores (means and standard deviations) for deep and surface approaches to learning, perceived stress score for the PT and the traditional cohorts at Time 1 and Time 2 are presented in Table 2. Independent samples t-tests showed no significant differences in either approaches to learning between PT and traditional cohorts at Time 1.

**Table 2:** Descriptive statistics for progress test (PT) and questionnaire scores

			Surface approach		Deep approach		Stress	
Cohort	Gender		Time 1	Time 2	Time 1	Time 2	Time 1	Time 2
PT	Female	Mean	9.72	9.87	19.16	19.03	16.79	18.75
	(*N* = 61)	SD	4.59	4.46	5.06	5.85	5.72	6.96
		Range	0–21	2–21	7–29	5–35	7–32	5–36
	Male	Mean	11.58	12.49	19.53	20.33	17.00	16.93
	(*N* = 43)	SD	5.71	5.90	5.32	5.70	6.59	6.30
		Range	1–27	6–33	4–29	4–32	4–36	3–28
Traditional	Female	Mean	11.32	11.39	19.34	19.39	16.20	20.21
	(*N* = 44)	SD	5.42	6.31	5.59	5.70	6.50	6.91
		Range	3–25	1–24	7–31	5–28	4–30	6–33
	Male	Mean	13.14	13.55	16.95	17.59	15.73	18.67
	(*N* = 22)	SD	5.91	6.05	4.08	4.83	6.27	6.47
		Range	3–25	3–28	10–24	8–25	7–27	4–29

At Time 1, students’ surface approaches to learning was significantly correlated with age (*r* = −.11, *p* < .05). At Time 2, both deep (*r* = .12, *p* < .05) and surface approaches to learning (*r* = −.15, *p* < .05) were significantly correlated with age. Gender also influenced approaches to learning and stress. Significant gender differences were found on surface approaches to study (*t* = −3.46, *p* < .05) and perceived levels of stress (*t* = 2.54, *p* < .05) at Time 1, and again on surface approaches to learning at Time 2 (*t* = −4.21, *p* < .05). Given these significant associations, gender and age were included in the main analyses.

At Time 1, the PT and traditional groups did not differ significantly from each other in terms of deep and surface approaches to learning, as well as the levels of perceived stress. Moreover, one-samples t-test showed that the average PSS scores for the PT cohort at Time 1 (*M* = 16.73, *SD* = 6.07) was significantly higher than the population norm adjusted for the similar age group (*M* = 14.4, *SD* = 6.2; Cohen et al., 1983), *t*(189) = 5.73, *p* < .001. Similarly, the average PSS for the traditional cohort at Time 1 (*M* = 15.88, *SD* = 6.40) was also significantly higher than the population norm, *t*(143) = 3.15, *p* < .05.

### Main analyses

#### Research question 1

Do PTs increase deep approaches to learning and/or decrease surface approaches to learning compared with traditional assessments?

**Fig. 1:**
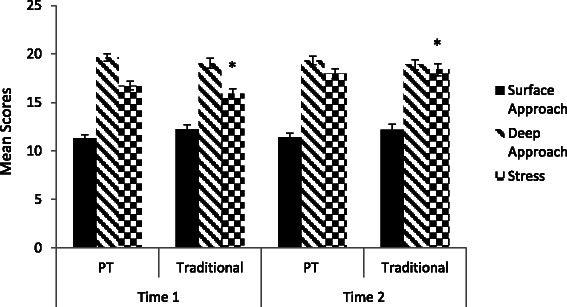
Approaches to learning and stress scores for PT and traditional group. Note. *p < .05

A 2 × 2 × 2 (approaches to learning × time × cohort) repeated-measures Analysis of Covariance (ANCOVA), while controlling for the effect of age and gender, showed no significant changes in either surface or deep approach scores from Time 1 to Time 2, regardless of cohorts. However, a pairwise comparison with Bonferroni adjustment showed that, in both cohorts, students scored significantly higher on deep approach to learning (*M* = 19.04, *SD* = .39) than surface approach to learning (*M* = 11.40, *SD* = .39). In addition, gender was a significant covariate across cohorts and time points (*F*(1, 166) = 4.00, *p* < .05, pη^2^ = .02); females scoring significantly lower on surface approaches to learning than males at both time point, whereas no gender differences were found for deep approaches to learning. See Fig. 1 for a comparison in surface and deep approaches to learning scores between PT and traditional groups.

#### Research question 2

Do PTs elicit less stress in students than traditional assessments?

A 2 × 2 (time × cohort) repeated-measures Analysis of Covariance (ANCOVA), while controlling for the effects of age and gender, showed no main effects for time or cohort on the levels of stress. However, there was a significant interaction between time and cohort (*F*(1, 164) = 5.86, *p* < .05, pη^2^ = .03). Follow-up paired samples t-tests showed a significant increase of stress from Time 1 (*M* = 15.94, *SD* = 6.37) to Time 2 (*M* = 19.70, *SD* = 6.76) for students in the traditional cohort (*t*(63) = 4.84, *p* < .05), whereas the levels of stress did not change significantly for students in the PT cohort. See Fig. 1 for a comparison in stress scores between PT and traditional groups.

#### Research question 3

Do deep or surface approaches to learning correlated with self-reported stress or PT scores?

The following analyses were based on students in the PT cohort only. As shown in Table 3, surface and deep approaches were negatively correlated at both time points. Surface approach was negatively correlated with PT scores at Time 1 but not at Time 2. The Level of perceived stress was positively correlated with surface approach score at both time points; stress was also negatively correlated with deep approach score at Time 1.

**Table 3:**
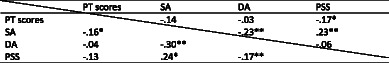
Zero-order correlations among Progress Test (PT) scores, approaches to learning and levels of perceived stress for Time 1 (below the diagonal line) and Time 2 (above the diagonal line)

1. *PT* Progress Test, *SA* surface approach, *DA* deep approach, *PSS* levels of perceived stress

2. *p < .05, **p < .01

Given these significant correlations, step-wise regressions were conducted separately for Time 1 and Time 2 to tease out the unique contribution of each factor of interest on PT scores. In the regression model, PT score was the dependent variable, student year in programme, gender, and age were entered at step 1 as the control variables. Surface approach, deep approach and perceived stress were entered at step 2 as the main predictors.

As shown in Table 4, both models showed good fit (adjusted R^2^ values ranged from 66 to 72 %). Students’ deep approach to learning scores and their perceived levels of stress did not significantly predict PT scores at either Time 1 or Time 2. In contrast, students’ year in the programme was a consistent predictor of PT scores at both time points, while the influence of age and gender on PT score was significant at Time 1 but decreased to non-significance at Time 2). Most importantly, students’ surface approach to learning score was a unique predictor of PT scores at both Time 1 and Time 2, above and beyond the influence of year in programme, age, and gender.

**Table 4:** Step-wise regressions to predict progress test scores at Time 1 and Time 2

	Step 1			Step 2		
Variables	B	β	*p*	B	β	*p*
Time 1: (*N* = 187)						
Year in programme	13.16	.78	< .001	13.05	.77	< .001
Gender	2.92	.08	< .05	3.60	.11	< .001
Age	.80	.15	< .001	.77	.15	< .05
Adjusted R^2^		.70				
F for ΔR^2^		148.68 (*p* < .001)				
Surface approach				-.36	-.12	<.05
Deep approach				.003	.001	ns
Stress				-.09	-.03	ns
Adjusted R^2^					.72	
F for ΔR^2^					3.75 (*p* < .05)	
Time 2: (*N* = 169)						
Year in programme	12.20	.80	<. 001	12.05	.79	< .001
Gender	-.05	-.002	ns	.64	.02	ns
Age	.22	.05	ns	.26	.05	ns
Adjusted R^2^		.66				
F for ΔR^2^		110.27 (*p* < .001)				
Surface approach				-.33	-.12	<.05
Deep approach				-.10	-.04	ns
Stress				-.04	-.02	ns
Adjusted R^2^					.67	
F for ΔR^2^					2.41 (*p* = .07)	

## Discussion

Over two time points, we did not find significant increases in deep approaches to learning or significant decreases in surface approaches to learning for students in the PT group, nor did we find any significant changes in either deep or surface approaches to learning for students in the traditional assessment group. PTs may have reduced stress for students, as students in the traditional group experienced significant increases in stress when they sat the traditional end of year exams, whereas the levels of stress did not increase significantly for students in the PT group. For students who sat PTs, their deep approach to learning score and perceived stress did not significantly predict their PT scores at either time point. In contrast, students’ surface approach to learning score was a unique negative predictor of PT scores, above and beyond the influence of year in programme, age, and gender, such that higher surface approach scores predicted lower PT scores at both time points.

Although it has been suggested that PT fosters a deep learning approach [3], we did not find significant changes in either deep or surface approaches over two time points for students participating in our survey who were involved in PT in our institution. This may be due to the fact that our medical students use both deep and surface approaches to learning and that a longer period of time is needed to change such established behaviour. Also, as our students are already showing higher levels of deep relative to surface approaches to learning, it may be difficult to detect any further increase in deep approaches to learning [32]. However, our measure of approaches to learning – Biggs’ Study Process Questionnaire [27] – is a reliable measure of deep and surface approaches to learning and thus should be sensitive to changes in behaviour. Future studies involving larger samples and data collection points over a longer time period are needed to confirm the current findings.

Consistent with the suggestion that low-stakes assessments should not elevate the levels of stress toward exam preparation [3], we found that stress remained relatively stable for students in the PT group between Time 1 and Time 2, whereas students in the traditional assessment group experienced significant increases in the levels of stress from Time 1 to Time 2, around the time when they were sitting end of year exams. Also consistent with previous literature we found medical students participating in our study to be more stressed than general population norms when using the Perceived Stress Scale [28]. Our findings suggest that PTs could be beneficial to students’ wellbeing compared with traditional high-stakes exams.

At both time points, surface approaches to learning significantly predicted students’ PT scores, above and beyond the effects of year in programme, age, and gender. Deep approaches to learning and the levels of perceived stress were not significantly related to PT scores at either time points. This adds to the inconsistent literature on the relationship between approaches to learning and students’ academic performance. The majority of previous research emphasised the influence of deep approaches to learning on student academic performance. Our research highlights the potential negative influence of surface approaches to learning on student academic achievement and calls for further research on how to discourage students from adopting surface learning strategies. Similar to previous studies [33], we found that approaches to learning are linked to students’ perceived stress such that higher levels of surface approach were linked to higher levels of perceived stress, whereas the opposite relationship was found at one time point between deep approach to learning scores and perceived stress. Future research could further explore whether stress mediates the link between approaches to learning and PT scores.

### Limitations

The response rate was 34–39 %, consistent with Nulty’s [34] review article reporting that response rates for online surveys were commonly in the vicinity of 33 %, but this does raise potential issues of how representative the sample is of the rest of the student population. However, survey respondents’ PT scores and demographics did not differ significantly from those who did not respond. There may be a range of reasons why student stress or their approaches to study might vary over time other than as a result of assessment experience, e.g. holiday periods, examination load, life events, and concurrent clinical attachments. Finally time-limited data collection does not allow comparisons on data collected at similar time points in consecutive years. However, this was the only chance for us to compare two cohorts in terms of the impact of different assessments on student learning and stress. We acknowledge there may be a systematic bias when comparing medical students in years 2 and 4 (cohort Progress Testing) with medical students in year 3 and 5 (cohort high-stakes assessment). Given that the students are measured cross-sectionally and not longitudinally, there is some risk that the students in years 3 and 5 have different learning experiences to those in years 2 and 4 and there is also an evident difference in terms of educational exposure. It is possible that this may be a confounding influence and future longitudinal study is needed to rule out this possible confounder.

## Conclusion

Changes to assessment regimes must always be evaluated for their impact on students’ learning outcomes and wellbeing. Our results do not support the theoretical claim that PTs drive a deep approach to learning. However, consistent with established theory, our results show that PTs appear to have somewhat reduced the examination stress of medical students, given that lower levels of stress were reported in the students undertaking PT. We also report a general negative effect of surface approaches to learning on PT scores, suggesting that aiming for an in-depth understanding of the medical curriculum may yield better results than cramming for examinations.

## Footnotes

1 PT1, the first Progress Test, was carried out in April 2013, prior to the commencement of the current project.
